# Force Transmission Between the Gastrocnemius and Soleus Sub-Tendons of the Achilles Tendon in Rat

**DOI:** 10.3389/fbioe.2020.00700

**Published:** 2020-07-17

**Authors:** Connor C. Gains, Janaina C. Correia, Guus C. Baan, Wendy Noort, Hazel R. C. Screen, Huub Maas

**Affiliations:** ^1^Institute of Bioengineering, School of Engineering and Materials Science, Queen Mary University of London, London, United Kingdom; ^2^Department of Human Movement Sciences, Faculty of Behavioural and Movement Sciences, Vrije Universiteit Amsterdam, Amsterdam Movement Sciences, Amsterdam, Netherlands

**Keywords:** Achilles tendon, force transmission, interfascicular matrix, shear, immunohistochemistry

## Abstract

The Achilles tendon (AT) is comprised of three distinct sub-tendons bound together by the inter-subtendon matrix (ISTM). The interactions between sub-tendons will have important implications for AT function. The aim of this study was to investigate the extent to which the ISTM facilitates relative sliding between sub-tendons, and serves as a pathway for force transmission between the gastrocnemius (GAS) and soleus (SOL) sub-tendons of the rat AT. In this study, ATs were harvested from Wistar rats, and the mechanical behavior and composition of the ISTM were explored. To determine force transmission between sub-tendons, the proximal and distal ends of the GAS and SOL sub-tendons were secured, and the forces at each of these locations were measured during proximal loading of the GAS. To determine the ISTM mechanical behavior, only the proximal GAS and distal SOL were secured, and the ISTM was loaded in shear. Finally, for compositional analysis, histological examination assessed the distribution of matrix proteins throughout sub-tendons and the ISTM. The results revealed distinct differences between the forces at the proximal and distal ends of both sub-tendons when proximal loading was applied to the GAS, indicating force transmission between GAS and SOL sub-tendons. Inter-subtendon matrix tests demonstrated an extended initial low stiffness toe region to enable some sub-tendon sliding, coupled with high stiffness linear region such that force transmission between sub-tendons is ensured. Histological data demonstrate an enrichment of collagen III, elastin, lubricin and hyaluronic acid in the ISTM. We conclude that ISTM composition and mechanical behavior are specialized to allow some independent sub-tendon movement, whilst still ensuring capacity for force transmission between the sub-tendons of the AT.

## Introduction

The Achilles tendon (AT) is the largest and strongest tendon in the human body. It forms a fundamental component of the musculoskeletal system, enabling everyday movements by bearing high loads and storing energy to reduce their energetic cost (Alexander, 1984; Komi, 1990; Fukashiro et al., 1995). In humans, the AT is exposed to forces exceeding 12 times bodyweight during running (Komi et al., 1992), and strains of up to 16% during a one-legged hop *in vivo* (Lichtwark and Wilson, 2005). It is estimated to store up to 35% of the total energy lost and regained during locomotion (Ker et al., 1987; Alexander, 1991). Such high strains mean that the AT functions markedly close to its failure properties (Wren et al., 2001), and is consequently vulnerable to injury. Tendinopathy of the AT is a highly debilitating condition. It accounts for up to half of all sports-related injuries (Kaux et al., 2011; Freedman et al., 2014; Lantto et al., 2015; Li et al., 2017), and a notable rise in the frequency of cases among the sedentary and aging populations is also reported over recent decades (de Jonge et al., 2011). Repetitive overload has been postulated as a major precursor for tendinopathy, but due to insufficient understanding of AT structure-function relationships, the causative mechanisms behind tendinopathy remain poorly understood (Wang et al., 2006; Riley, 2008).

**FIGURE 1:**
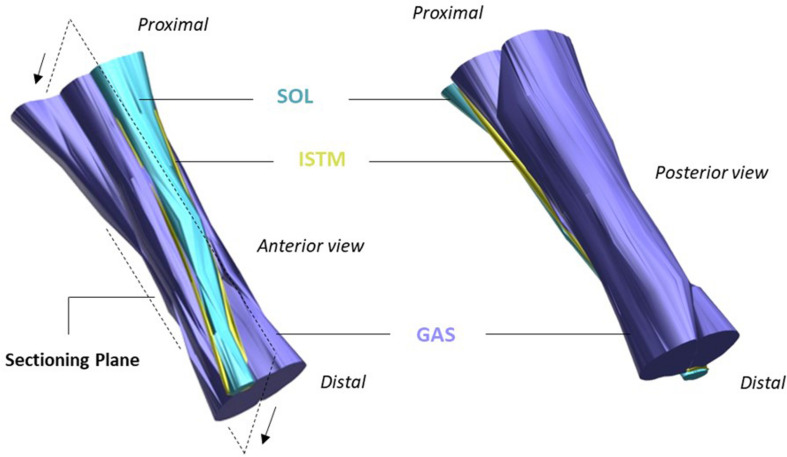
Structure of the rat Achilles tendon (AT). The gastrocnemius (GAS, purple), and soleus (SOL, blue) sub-tendons are bound together by the inter-subtendon matrix (ISTM, yellow). The plane in which samples were sectioned for histology/immunohistochemistry is shown, with arrows indicating the cutting direction. Adapted from Finni et al. (2018).

Tendons are hierarchical fiber-composites, in which collagen molecules aggregate to form highly ordered sub-units of increasing diameter, up to the whole tendon level. At the larger length-scales, the collagenous sub-units are interspersed with a highly hydrated proteoglycan-rich matrix (Thorpe et al., 2013a). The hierarchical structure of tendon has long been established (Kastelic et al., 1978), yet discrepancies remain prevalent in the literature regarding the correct nomenclature to define the sub-units at each length scale. This is compounded by the differences in tendon hierarchical organization seen between functionally distinct tendons, and those seen across species of different sizes. Fascicles have typically been considered to be the largest sub-unit in tendon beneath the whole tendon level, but the AT is an exception, as the tendon derives from the three distinct muscles bellies of the triceps surae complex (Bojsen-møller and Magnusson, 2015). As such, the AT includes an additional macro level between fascicles and tendon, where the elements of the AT arising from each muscle belly of the triceps surae form separate soleus (SOL) and gastrocnemius (GAS) “sub-tendons” (Handsfield et al., 2016). As the GAS and SOL sub-tendons of the AT descend from their respective muscles, they laterally rotate and insert onto the calcaneal bone (Edama et al., 2015). Along their given lengths, the AT sub-tendons are bound together by the inter-subtendon matrix (ISTM; Figure 1). Although the sub-tendons are tightly fused together by the ISTM, anatomical studies have shown the sub-tendons to differ morphologically, allowing them to be macroscopically distinguished and dissected (Szaro et al., 2009; Edama et al., 2015; Finni et al., 2018).

Recent human studies have demonstrated non-uniform displacements in the AT, revealing that the anterior portion of the AT experiences greater displacements than the posterior portion during passive ankle motions (Arndt et al., 2012), eccentric plantarflexor contractions (Slane and Thelen, 2014), and isometric contractions, irrespective of the knee angle or level of force produced (Bogaerts et al., 2018). While these studies in human subjects were not able to attribute the non-uniform behavior to specific sub-tendons, recent work has shown that the SOL sub-tendon in the rat AT experiences significantly different displacements and strains than the lateral GAS sub-tendon during isometric contractions (Finni et al., 2018; Maas et al., 2020), indicating non-uniform deformations of the AT sub-tendons.

Several factors are likely to be responsible for the non-uniformities observed within the AT. While the specialized macroscopic design of AT may play an important role (Bojsen-møller and Magnusson, 2015; Edama et al., 2015; Pękala et al., 2016), the ISTM at the microscopic level is also highly likely to contribute. The matrix between fascicles (interfascicular matrix; IFM) has been shown to enable fascicle sliding in both human and equine tendons, a crucial component of enabling tendon extension and recoil (Thorpe et al., 2012, 2013b, 2015b; Franz et al., 2015; Franz and Thelen, 2016). In the same manner, the ISTM may permit the relative sliding between the AT sub-tendons to enable non-uniform AT loading. However, the role of ISTM in enabling sub-tendon sliding or serving as a pathway to transmit forces between sub-tendons and distribute load through the AT is currently unknown. This may have significant implications, as the transmission of force across sub-tendons may act to distribute loads across a greater cross-sectional area (CSA), thereby reducing peak stresses to mitigate the risk of tissue overload (Maas and Finni, 2018).

Studies attempting to characterize the distribution of proteins within the different compartments of tendon, particularly in the non-collagenous matrix at the larger length scales, are limited. In the horse superficial digital flexor tendon (SDFT), the IFM has a distinct proteomic profile compared to that of the surrounding fascicles, displaying a greater number of proteins (Thorpe et al., 2016b). Further evidence suggests differences exist in the cell populations present in the IFM and within fascicles, where those in the former appear morphologically more rounded, and reside at a significant greater density (Thorpe et al., 2015a). Immunohistochemical studies have shown both lubricin (Sun et al., 2006, 2015; Funakoshi et al., 2008; Thorpe et al., 2016a), and elastin (Smith et al., 2011; Grant et al., 2013; Godinho et al., 2017) to be highly localized to the IFM, where it is suggested they may contribute to the specialized composition of IFM which enables its highly elastic mechanical behavior. The protein distribution of the ISTM in the AT, however, remains poorly characterized, preventing further correlation between its specialized composition and distinct mechanical properties.

The aims of the current study were (i) to investigate to what extent the ISTM facilitates relative sliding between sub-tendons and/or serves as a pathway for force transmission to distribute forces through the AT, (ii) to assess the mechanical behavior of the ISTM, and (iii) to characterize the composition of the ISTM. We hypothesized that the ISTM provides a mechanical linkage between GAS and SOL sub-tendons, with mechanical behavior that enables some inter-subtendon sliding, whilst also ensuring force transmission between sub-tendons. We also hypothesized that the distribution of proteins, and organization of cells would differ between the ISTM and sub-tendon compartments.

## Materials and Methods

### Animals

For mechanical characterization, a single hindlimb from 10 Wistar rats (7 female, 3 male, body mass 240–360 g) was excised and frozen (−80°C) immediately after sacrifice with an intracardial overdose injection of pentobarbital sodium (Euthasol 20%), along with double-sided pneumothorax. For immunohistochemical studies, one hindlimb from eight Wistar rats (sex unknown, body mass 200–220 g) was excised and prepared for embedding immediately after an overdose of isoflurane and decapitation. All procedures were in strict agreement with the regulations set out in EU law and approved by local university ethical committees, with limbs taken as left-over tissue from other unrelated studies.

### Sample Preparation

**FIGURE 2:**
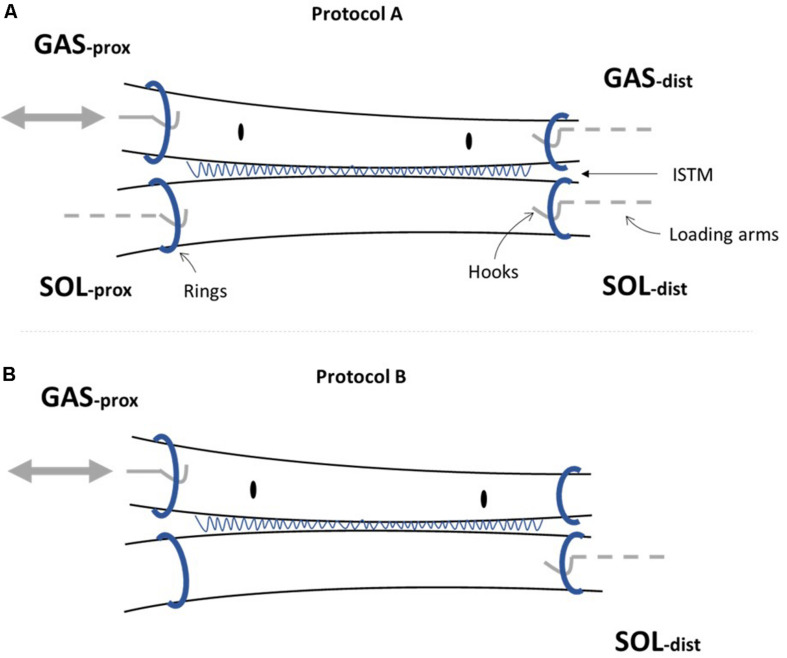
Achilles tendon (AT) sample preparation for mechanical testing. Metal rings were attached to the proximal (prox) and distal (dist) ends of the gastrocnemius (GAS) and soleus (SOL) sub-tendons to secure samples to the rig set-up. The sub-tendons are bound together by the inter-subtendon matrix (ISTM). **(A)** For protocol A [(i) and (ii)], all four ends of the AT sample were attached to the loading arms with force transducers (GASprox,GASdist, SOLprox and SOLdist), where a servo-motor was additionally connected to the GASprox, allowing the tendon to be stretched at a given speed and length according to the loading protocol. **(B)** To determine the mechanical behavior of the inter-subtendon matrix (ISTM) in protocol B, only the proximal gastrocnemius (GASprox) and distal soleus (SOLdist) rings remained connected to the set-up, with displacement still applied via the motor connected to the GASprox to load the ISTM in shear. Black dots indicate the sutured markers on the GAS sub-tendon used for internal strain analysis.

On the day of mechanical testing, hindlimbs were allowed to thaw at room temperature. To obtain access to the triceps surae muscle complex, the limbs were shaved, followed by removal of the skin and biceps femoris muscle. The AT was then exposed by removing all remaining connective and fat tissues surrounding the tendon structure. The GAS and SOL muscles were identified and separated down to the muscle-tendon junction to allow preparation of the proximal ends of the GAS and SOL sub-tendons. Distal separation of GAS and SOL sub-tendons was achieved by gently dissecting through a small region of the ISTM at the calcaneal insertion. Metal rings were then attached to the proximal and distal ends of the GAS and SOL sub-tendons via threaded knots to allow connection to the mechanical testing set-up (Figure 2A). The distance between rings is referred to as the grip-to-grip distance hereafter. Marker knots were sutured onto the surface of the GAS sub-tendon to assess internal strains within the mid-portion of the GAS sub-tendon (the middle 1/3 of the total tendon length).

AT samples intended for immunohistochemistry were dissected immediately after sacrifice, embedded longitudinally (*n* = 5–8) in optimal cutting temperature compound (OCT) and snap-frozen in hexane cooled on dry ice. Cryosections covering the full length of the AT were cut 15 μm thick, transferred to polylysine slides, and stored at −80°C until required for staining (Table 1).

**TABLE 1:** Details of the primary antibodies used for immunohistochemical staining.

**Antibody**	**Host species**	**Mono/polyclonal**	**Epitope recognized**	**Concentration**	**Manufacturer (product code)**
Decorin	Rabbit IgG	Polyclonal	Core protein	1:500	Atlas Antibodies (HPA003315)
Collagen type III	Rabbit IgG	Polyclonal	Core protein	1:150	Abcam (ab7778)
Elastin	Rabbit IgG	Polyclonal	Core protein	1:50	Abcam (ab21610)
Lubricin	Mouse IgG	Monoclonal	C-terminal domain	1:50	MD Bioproducts (1045015)

### Mechanical Testing Set-Up

The AT samples were secured within the loading rig by connecting the metal rings attached to the proximal and distal ends of the GAS and SOL sub-tendons to hooks on the rig loading arms (Figure 2). Each loading arm was linked to a force transducer (ALPHA load beam transducer, 25 N maximum capacity, maximum output error <0.1%, compliance 0.0162 mm/N, BLH Electronics Inc.), mounted on a single-axis micro-positioner. The force transducer linked to the loading arm at the proximal end of the GAS sub-tendon was mounted on a servo-motor (MTS50C-Z8, T-Cube Servo-motor driver, Thorlabs, Cambridgeshire, United Kingdom), to impose displacements to the proximal end of the GAS sub-tendon (Figure 2). A video camera (Panasonic HC-V720, resolution: 1 pixel ∼0.03 mm) was positioned above the loading set-up to record the grip-to-grip distance and strains between suture markers in the GAS sub-tendon, with a millimeter ruler positioned besides the AT samples for calibration.

### Mechanical Testing Protocols

**FIGURE 3:**
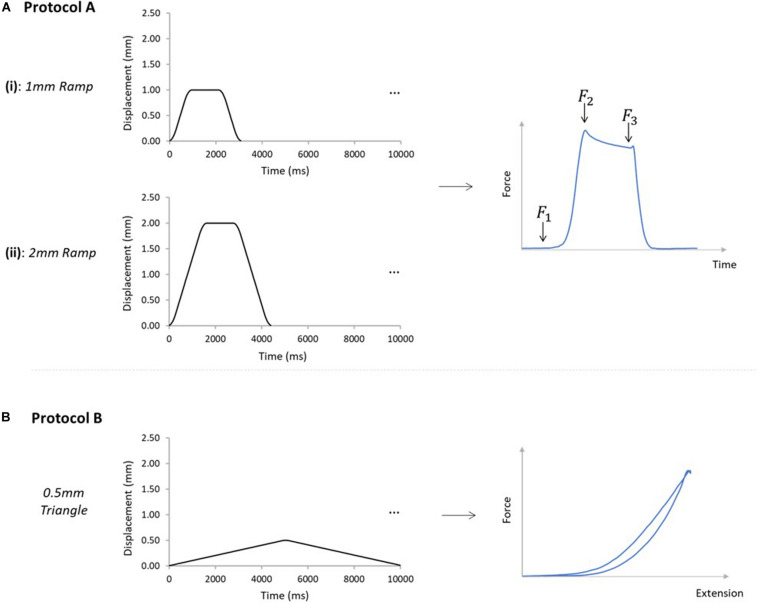
Mechanical testing protocols applied to AT samples. **(A)** Samples were subjected to protocols A [(i) and (ii)], and B, in order on all analyzed samples. Protocol A(i) applied a 1mm ramp protocol to the GASprox, at an average speed of 1.1 mm/s for 10 cycles. Protocol A(ii) applied using the same displacement waveform but to a peak displacement of 2 mm, at an average speed of 1.3 mm/s for 10 cycles. Protocol A was devised to investigate force transmission between the sub-tendons, where the force before the stretch (*F*_*1*_), peak force (*F*_*2*_), and force in the steady state (*F*_*3*_) were analyzed. **(B)** Protocol B was applied with only the GASprox and SOLdist attached to their respective loading arms and force transducers, where a 0.5 mm triangular waveform (0.1 mm/s, 10 cycles) was imposed to the GASprox to load the inter-subtendon matrix in shear and assess its mechanical behavior.

Two different loading protocols were applied (Figure 3). Protocol A was designed to assess force transmission between the GAS and SOL sub-tendons, simulating two different levels of physiological GAS muscle activation and muscle belly shortening, while the SOL muscle length remains largely unchanged. Such conditions reflect the preferential recruitment of the GAS muscle in the triceps surae, as observed *in vivo* during isometric plantarflexor contractions at varying knee joint angles (Cresswell et al., 1995) and maximal knee extension (Bojsen-møller et al., 2004). Protocol B was designed to indirectly determine the mechanical behavior of ISTM. Each sample was tested through protocols A and B in this order, and all tests were performed at room temperature.

For protocol A, preloads of 0.025 and 0.015 N were applied to the GAS and SOL sub-tendons respectively. These preloads were designed to account for the differences we measured in the GAS and SOL sub-tendon cross sectional areas (Table 2), and to ensure consistent pre-stress conditions. Protocol A(i) applied a 1mm ramp displacement to the loading arm attached to the proximal end of the GAS sub-tendon at an average speed of 1.1 mm/s (0.9 s:1.3 s:0.9 s stretch:hold:release) for 10 cycles (Figure 3A). Directly after completing protocol A(i), the pre-loads were re-applied, and protocol A(ii) was initiated, imposing a 2 mm stretch at an average speed of 1.3 mm/s (1.6 s:1.2 s:1.6 s stretch:hold:release) to the loading arm for 10 cycles (Figure 3A). On completion of protocol A, the hooks attached to the distal GAS and proximal SOL were disconnected from the mechanical set-up, thus leaving only the proximal GAS and distal SOL secured to the loading arms (Figure 2B). Under this configuration (protocol B), displacement of the proximal GAS loaded the ISTM in shear. A pre-load of 0.015 N was applied to the sample to remove any slack. Samples were then subjected to 10 stretch cycles of 0.5 mm using a triangular waveform at a speed of 0.1 mm/s (5.0 s:5.0 s stretch:release) (Figure 3B). All exposed tissues were regularly irrigated with phosphate-buffered saline (PBS) throughout preparation to maintain sample hydration.

**TABLE 2:** Forces measured at the proximal and distal ends of the GAS and SOL sub-tendons in the 10th cycle of the 1 mm [protocol A(i)] and 2mm [protocol A(ii)] stretch imposed at the proximal GAS.

	**Protocol A(i)**				**Protocol A(ii)**			
	**GASprox**	**GASdist**	**SOLprox**	**SOLdist**	**GASprox**	**GASdist**	**SOLprox**	**SOLdist**
*F*_*1*_ (N)	0.0020.001	0.0010.001	0.0020.002	0.0010.001	0.0000.002	0.0010.002	0.0030.003	0.0000.001
*F*_*2*_ (N)	0.3640.218	0.2850.215	0.0080.008	0.0840.051	1.4180.825	1.0810.759	0.0070.007	0.3410.163
*F*_*3*_ (N)	0.3240.203	0.2530.198	0.0050.007	0.0750.046	1.2850.774	0.9820.715	0.0040.006	0.3060.149
Stress relaxation (%)	34.96.8	32.56.5	49.420.4	35.47.5	44.25.4	45.510.8	57.721.9	43.85.4

Total AT length (mm)		8.661.13						
GAS CSA (mm^2^)		1.190.26						
SOL CSA (mm^2^)		0.600.44						

*Stress relaxation measured at each load cell over all 10 cycles of protocol A(i) and A(ii). Geometric properties of the samples measured after mechanical testing, including the total AT length, and the cross-sectional area (CSA) of the GAS and SOL sub-tendons.*

After mechanical testing, the sub-tendons of the AT were manually separated, to assess whether the GAS and SOL sub-tendons had been correctly isolated. Samples where the sub-tendons were entirely separated along their lengths (termed “isolated” samples) were analyzed as one group. In samples where a fiber remained (termed non-isolated samples), that fiber may contribute toward force measurements if running from proximal GAS to distal SOL, or be subject to no load if running from proximal SOL to distal GAS. Non-isolated samples with a fiber running from the proximal SOL to the distal GAS were included in the analysis as a secondary group. A comparison of data from isolated and the non-isolated samples confirmed no impact on force measurements (four isolated samples; six non-isolated isolated; comparison of forces exerted at the sub-tendons distally, *p* = 0.171), hence data from both conditions were pooled in the analysis. In non-isolated samples where fibers ran from the proximal GAS to the distal SOL, or across both directions, fibers would undoubtedly introduce significant errors to the estimation of force transmission via the ISTM, so samples were excluded from further analysis.

After manual separation, the total tendon sample length and the CSA of the GAS and SOL sub-tendons were measured. The samples lengths were measured through analysis of the video recordings. To measure CSA, the sub-tendons were cut transversely, imaged with a microscope camera (Bresser MicroCam 5.0), and measured using ImageJ (Schneider et al., 2017).

### Data Recording and Analysis

Force signals were sampled at 1000 Hz, processed through a low-pass Butterworth filter with a cut-off frequency of 10 Hz, and stored for analysis. Images of the AT during loading were recorded at 50 frames/s (Panasonic HC-V720).

#### Protocol A [(i) and (ii)]

The force prior to the stretch (*F*_*1*_), the peak force at the end of stretch (*F*_2_), and the steady state force at the end of the hold phase (*F*_*3*_), were measured from all four force transducers across all 10 cycles (Figure 3A). The percentage reduction in the peak force was calculated across the 10 cycles, providing a measure of the stress relaxation behavior during pre-conditioning (hereafter referred to as “stress relaxation”). The steady state force (*F*_*3*_) of cycle 10 was used to compare force values across the four force transducers, and, thus, assess force transmission between sub-tendons. Percentage differences between the forces at the proximal and distal ends of each sub-tendon were calculated by dividing the difference between proximal and distal steady-state forces of each sub-tendon in cycle 10, by the mean of the two force values. Data obtained from 10 samples was analyzed using a custom-written MATLAB code (R2018a, MathWorks, Natick, MA, United States). Strains relating to the GAS sub-tendon were assessed during loading, through analysis of the video recordings using Tracker (Tracker Video Analysis and Modeling Tool, 5.0.7, D. Brown and Cox, 2009). External strains were calculated through analysis of the grip-to-grip length changes between the proximal and distal GAS rings. Internal strains were assessed through the mid-portion length changes, measured between the suture markers. Video recordings from seven samples were available for external strain analysis. Internal strains were calculated from five samples, as it was not possible to suture markers on the SOL sub-tendon of all samples. Values for the internal strains were compared with the applied strain, and the external strains in the 10th cycle of each ramp protocol.

#### Protocol B

For each stretch cycle, hysteresis was calculated as the difference in area between the loading and unloading curves, while total hysteresis was calculated as the difference between the loading curve of cycle 1 and the unloading curve of cycle 10. Stress relaxation was calculated as the percentage reduction in the peak force across the 10 cycles. Maximal stiffness was calculated by measuring the continual stiffness over each cycle from the slope of the force-displacement data (500 data points per cycle), and taking the maximum value from cycles 1 to 10. Values were then compared from cycles 1 to 10 to assess changes. Data were obtained from nine samples, as for one experiment Protocol B was not imposed due to a technical issue.

To ensure the ISTM was being loaded in shear, the grip-to-grip distances were used to measure the external strains in each of the GAS and SOL sub-tendons and also the relative movement between the two sub-tendons using the Tracker software. ISTM strains were calculated from the distance between the proximal GAS and distal SOL rings. Video recordings from seven samples were available for external strain analysis.

### Immunohistochemistry

Immunohistochemistry was used to assess the distribution of several proteins (decorin, collagen type III, elastin, and lubricin) in the ISTM and sub-tendon (ST) regions of the AT. The choice of proteins for staining was guided by the low stiffness, highly elastic behavior we observed in the ISTM from Protocol B. Sections were allowed to thaw at room temperature, before fixing in ice-cold acetone (−20°C) for 10 min. Slides were then washed three times in tris-buffered saline (TBS), and to improve permeability, pre-treated with either Chondroitinase ABC (0.2 U/ml) for 1 h (decorin), or hyaluronidase [4800 U/ml in PBS containing protease inhibitor cocktail (complete Mini, Roche)] overnight (collagen type III and elastin) at room temperature. Two further washes in TBS followed, before sections were incubated with blocking buffer, consisting of 10% serum in TBS + 0.1% bovine serum albumin (BSA) for 2 h at room temperature. Slides were then drained, and primary antibodies diluted in blocking buffer (Table 1) applied to sections and incubated overnight at 4°C. The slides were drained the following day and washed twice with TBS, before sections were incubated with 0.3% H_2_O_2_ in TBS for 15 min at room temperature to inhibit endogenous peroxidase activity. Directly after decanting, slides were incubated with secondary antibodies (1:50 in 10% serum in TBS + 0.1% BSA) for 1 h at room temperature. Sections were then rinsed three times in TBS, before the staining was developed using 3,3’-diaminobenzidine (DAB). Sections were counterstained using Gill’s hematoxylin, dehydrated in increasing concentrations of ethanol and xylene, and coverslipped using DPX mountant. Negative controls included sections incubated with (i) no primary antibody, to check for non-specific binding of the secondary antibody and (ii) a non-specific rabbit IgG antibody at the same concentration as elastin (highest concentration used for all antibodies raised in rabbit), to check for non-specific binding of the primary antibodies. The non-specific rabbit IgG was incubated on sections pre-treated with chondroitinase ABC and hyaluronidase (on separate sections) to validate non-specific binding of all primary antibodies. No staining was observed in any of these controls.

### Immunohistochemistry for Lubricin

Sections were allowed to thaw at room temperature, before fixing in 10% neutral buffered formalin for 5 min. Slides were then washed three times in PBS containing 3% Triton X-100, followed by blocking in 10% serum in TBST (TBS containing 0.5% Triton X-100) + 0.1% BSA (blocking buffer) for 2 h at room temperature. Samples were then incubated with lubricin monoclonal antibody diluted in blocking buffer overnight at 4°C. Sections were washed three times in TBST, and treated with 3% H_2_O_2_ for 1 h at room temperature to quench endogenous peroxidase activity. The samples were incubated with a biotinylated horse anti-mouse (rat absorbed) secondary antibody (1:50 in blocking buffer) for 1 h at room temperature, followed by incubation with avidin–biotin complex (ABC) reagent (Vectastain^®^ Elite ABC HRP Kit, Vector laboratories, United Kingdom) for 1 h at room temperature. After three washes in PBS, staining was developed with DAB. Sections were then dehydrated in increasing concentrations of ethanol and xylene, and coverslipped using DPX mountant. No counterstaining was performed on sections stained for lubricin, due to previous reports indicating the presence of intracellular lubricin in tendon (Sun et al., 2015; Thorpe et al., 2016a). Negative controls included sections incubated with (i) no primary antibody, to check for non-specific binding of secondary antibody, (ii) no primary or secondary antibody, to check for non-specific binding of the ABC reagent, and (iii) a non-specific mouse IgG antibody at the same concentration as the primary antibody, to check for non-specific binding of the primary antibody. No staining was observed in any negative controls.

### Histochemistry for Hyaluronic Acid

Histochemical staining was used to assess the distribution of hyaluronic acid (HA) in the ISTM and ST regions of the rat AT. Sections were allowed to thaw at room temperature, before fixing in ice-cold acetone (−20°C) for 10 min. Negative controls for HA were washed three times in TBS, and pre-digested with 30U of hyaluronidase (from *Streptomyces hyalurolyticus*, Sigma H1136) overnight at room temperature, before proceeding with the staining procedure. Slides were washed three times in TBS, followed by blocking in 10% serum in TBS + 0.1% BSA for 2 h at room temperature. Sections were then drained, and incubated with biotinylated HA binding protein (bHABP, 1:100) overnight at 4°C. The slides were drained the following day and washed twice with TBS, before sections were incubated with 0.3% H_2_O_2_ in TBS for 15 min at room temperature to quench endogenous peroxidase activity. After two further washes in TBS, sections were incubated with the ABC reagent (Vectastain^®^ Elite ABC HRP Kit, Vector laboratories, United Kingdom) for 1 h at room temperature. Three washes in TBS followed, before the staining was developed using DAB, counterstained using Gill’s hematoxylin, dehydrated in increasing concentrations of ethanol and xylene, and coverslipped using DPX mountant. An additional negative control was included which involved sections incubated without the bHABP, to check for non-specific reactions of the ABC reagent. No staining was observed in all negative controls.

### Histology

To assess the general structure and cellular organization of the ISTM and sub-tendon regions in the rat AT, longitudinal sections were stained with hematoxylin and eosin (H&E). Once sections had been thawed at room temperature, and fixed with ice-cold acetone (−20°C) for 10 min, slides were stained with H&E using the standard staining procedure (Stevens and Bancroft, 1990) and mounted.

### Image Acquisition

Two to three sections from each sample were stained with each of the five stains. Sections were imaged using a Leica DMIL light microscope at low magnification (×10), and high magnification (x20). To ensure the ISTM was within the field of view of all images, sections were first visualized under phase contrast enabling identification of the ISTM region without bias (Figure 6A).

### Image Scoring

Semi-quantitative methods were adopted to assess the distribution of proteins in the sub-tendons and ISTM of the AT. Two independent assessors scored the intensity of staining in immunohistochemical images, by grading the staining intensity in the sub-tendons and ISTM regions separately from 0 to 5, where 0 indicates no staining, and 5 represents very intense staining. The two assessors also graded cellularity, and the nuclear shape of cells within ISTM and sub-tendon regions using the H&E stained sections. For cellularity, images were graded from 0 to 5, where 0 indicates no cells present, and 5 indicates a very high number of cells. Nuclear shape was graded in each image from 0 to 5, where 0 indicates a highly elongated, spindle-like morphology, and 5 represents a rounded, circular nuclear shape. Inter-observer variability was assessed through linear weighted Kappa statistics (Viera and Garrett, 2005), using an online software tool^1^. Scorers were asked to grade every image twice to allow the calculation of intra-observer variability, where images were re-arranged on the second occasion to prevent bias scoring. Intra-observer variability was also calculated using linear weighted Kappa statistics.

### Statistics

Normality of all data sets was first determined using a Shapiro–Wilk test, after which non-parametric statistics was adopted for protocol A data, and parametric statistics for protocol B and immunohistochemical data. Wilcoxon rank sum tests were used to test for differences between steady-state forces across the GAS or SOL sub-tendons. A Mann Whitney test was conducted to assess differences in the steady-state forces at the distal end of the GAS and SOL sub-tendons of isolated and non-isolated samples. To assess the effects of cycle number on the ISTM mechanical behavior, a one-way analysis of variance (ANOVA) was performed. To assess the differences in staining intensity between the sub-tendon and ISTM regions, a one-way nested ANOVA was used. Statistical analysis was performed using GraphPad Prism 8 (GraphPad, Inc., San Diego, CA, United States), unless stated otherwise. Statistical significance was defined as *p* < 0.05. All data were displayed as mean ± SD, unless stated otherwise.

## Results

### Force Transmission Between the GAS and SOL Sub-Tendons

The grip-to-grip length changes (external strain) imposed on the GAS sub-tendon during the last cycle of protocol A(i) was 0.45 ± 0.25 mm (7.8% strain). Mid-portion length changes (internal strains) were substantially smaller (3.7%). The same behavior was true for protocol A(ii), where 0.83 ± 0.27 mm grip-to-grip lengthening (13.9% external strain) was measured in the GAS sub-tendon in the 10th cycle of the 2 mm stretch, accompanied by mid-portion length changes of 5.8% internal strain, suggesting non-uniform strain distributions along the GAS sub-tendon in both loading protocols.

**FIGURE 4:**
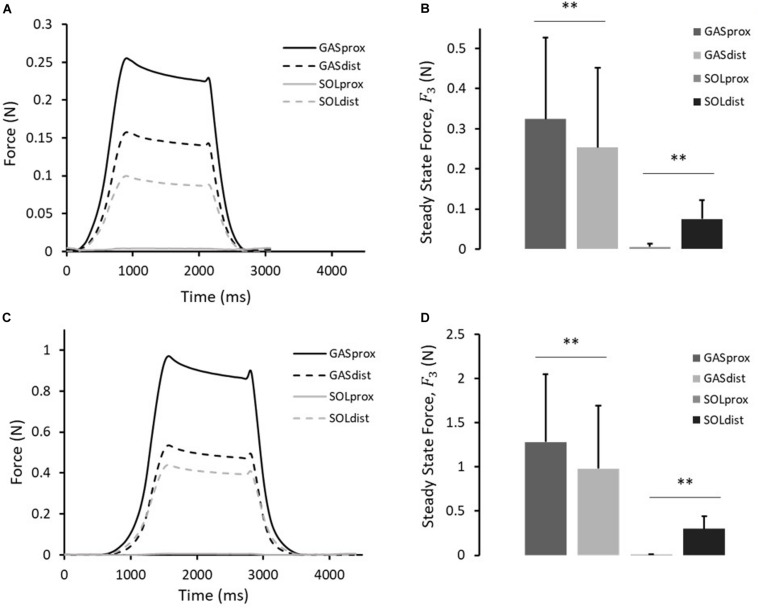
**(A)** Typical force response, and **(B)** the steady-state force, *F*_*3*_, measured at the proximal and distal ends of the GAS and SOL sub-tendons in 10th cycle of protocol A(i). **(C)** Typical force response, and **(D)** the steady-state force, *F*_*3*_, measured at the proximal and distal ends of the GAS and SOL sub-tendons in 10th cycle of protocol A(ii). Data presented as mean ± SD. Significant differences: **p < 0.01.

A typical force response of the proximal and distal ends of the GAS and SOL sub-tendons in the 10th cycle of protocol A(i), 1mm stretch, is presented in Figure 4A. The values for the force prior to stretch, (*F*_1_), peak force at the end of stretch (*F*_2_), steady state force at the end of the hold phase (*F*_3_), and percentage stress relaxation from cycle 1 to 10 for each load cell are presented in Table 2. With respect to the GAS sub-tendon, the forces exerted at both ends increased when subjected to the 1 mm stretch. However, the steady state force was significantly greater at the proximal end (*p* = 0.002; difference = 0.07 N, 31%) than the distal end (Figure 4B). The SOL sub-tendon similarly demonstrated significant differences between the steady state force at the proximal and distal ends, but in the opposite direction, i.e., greater force was measured at the distal end (*p* = 0.002; difference = 0.07 N, 173%) than at the proximal end (Figure 4B).

A typical force response from the four ends of the AT samples in the 10th cycle of protocol A(ii), 2 mm stretch, is presented in Figure 4C. The values for *F*_*1*_, *F*_*2*_, *F*_*3*_, and percentage stress relaxation from cycle 1 to 10 for each load cell are presented in Table 2. As seen during protocol A(i), the steady state force measured at the proximal end of the GAS sub-tendon was significantly higher (*p* = 0.002; difference = 0.30 N, 27%) than at the distal end, whilst a greater force was measured at the distal end of the SOL sub-tendon (*p* = 0.002; difference = 0.16 N, 194%) compared to proximal end (Figure 4D).

### Mechanical Behavior of the ISTM

**FIGURE 5:**
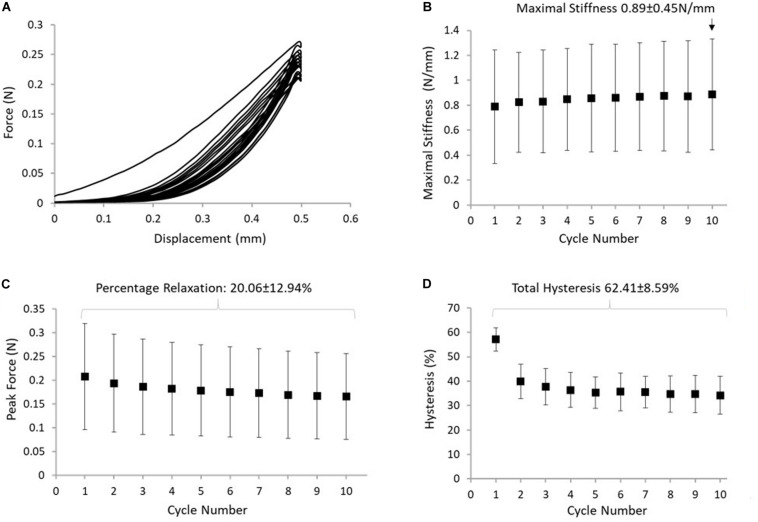
**(A)** Typical response of the ISTM to protocol B. **(B)** The maximum stiffness reached in the ISTM in each cycle of protocol, with the highest value measured in cycle 10 indicated above the data point. **(C)** The reduction in peak force, with the stress relaxation calculated over all 10 cycles indicated above the data points. **(D)** Hysteresis measured in the ISTM across all 10 cycles, with the total hysteresis over protocol B indicated above the data points. Data presented as mean ± SD.

Displacement of the proximal GAS and distal SOL rings was compared with applied displacement of the loading arm, confirming that the applied deformation was transferred to the ISTM during each loading cycle of the test. Changes in the distance between the proximal GAS and distal SOL in the 10^*th*^ cycle of protocol B measured 0.39 ± 0.06 mm. Strains in the sub-tendons during protocol B were found to be negligible (<0.6% strain), confirming the ISTM was exclusively strained in shear. Typical force-displacement curves for the 10 cycles of protocol B are presented in Figure 5A. We observed 57.1 ± 4.7% hysteresis in the first cycle, which decreased to 34.2 ± 7.7% in the 10th cycle. Over all 10 cycles, the total hysteresis was 62.4 ± 8.5% (Figure 5D). Hysteresis significantly changed from cycles 1 to 2 (*p* < 0.001), but analysis of cycles 2 to 10 showed an insignificant effect of cycle number on hysteresis (*p* = 1.000). Analysis of the early stages of stress relaxation showed the percentage force reduction over all 10 cycles was 20.1 ± 12.9% (Figure 5C), and the peak force did not significantly change with cycle number (*p* = 1.000). With respect to ISTM stiffness, a slight, but insignificant (*p* = 1.000) increase was observed over the 10 cycles, with the maximal stiffness in the 10th cycle measuring 0.89 ± 0.45 N/mm (Figure 5B).

### General AT Structure and ISTM Histology

**FIGURE 6:**
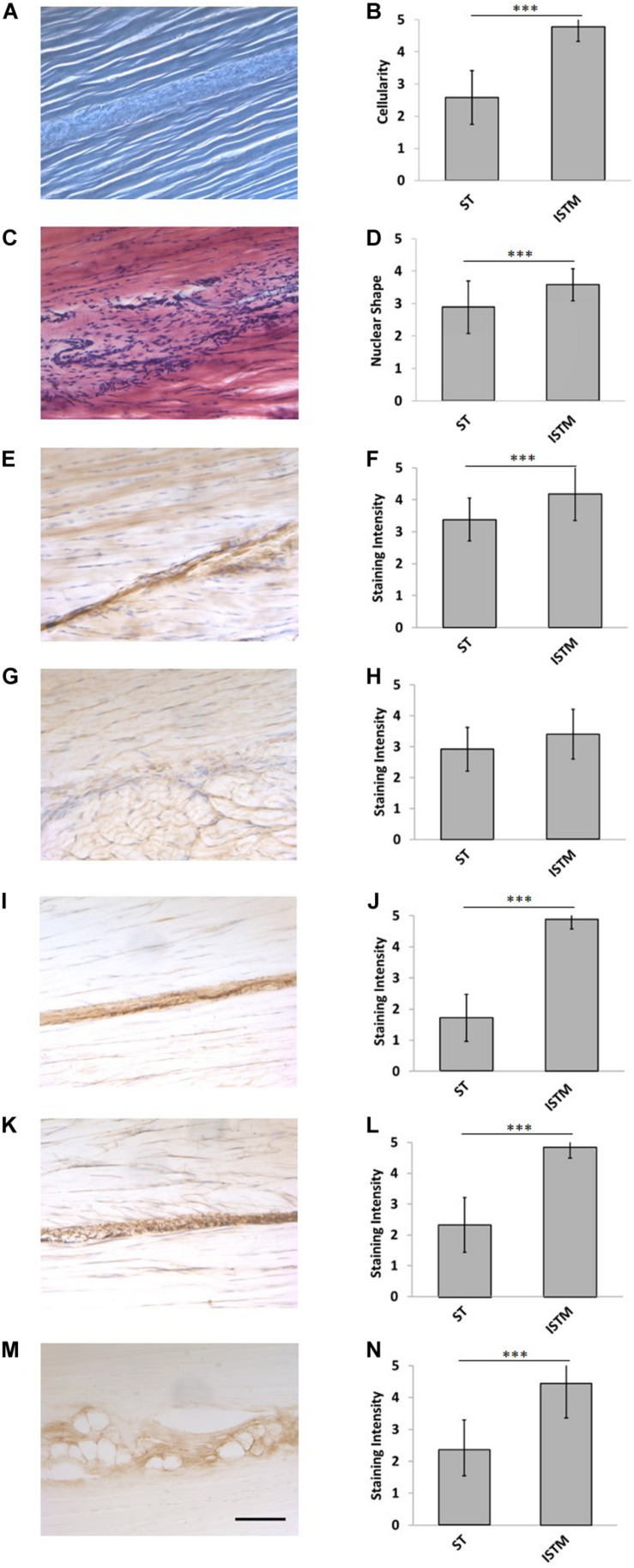
Representative histology and immunohistochemistry. **(A)** Histological section of the rat Achilles tendon imaged under phase contrast, demonstrating the GAS and SOL sub-tendon (ST) regions are bound together by the inter-subtendon matrix (ISTM). **(C)** H&E staining. There was a significant difference in the panel **(B)** celluarity, and **(D)** nuclear of shapes between the ISTM and sub-tendons. **(E)** Representative histochemical staining for HA. Representative immunohistochemical staining for **(G)** decorin, **(I)** collagen type III, **(K)** elastin, and **(M)** lubricin. Staining intensity for **(F)** HA, **(J)** collagen type III, **(L)** elastin, and **(N)** lubricin was significantly greater in the ISTM. Staining intensity for **(H)** decorin was not significantly different between regions. Scale bar: 100 μm. Data are displayed as mean ± SD.***p < 0.001.

Representative H&E staining of the AT is presented in Figure 6B. There was a significantly higher cellularity localized to the ISTM, than within sub-tendons (*p* < 0.001; Figure 6C). The nuclear shape of cells within ISTM and sub-tendons were also significantly different (*p* < 0.001), where nuclei in the ISTM were significantly rounder (Figure 6D).

### Decorin Distribution

Typical images of decorin staining are presented in Figure 6G. There were no significant differences in staining intensity between the ISTM and sub-tendon regions (*p* = 0.081; Figure 6H), with decorin appearing throughout the AT structure.

### HA Distribution

Typical images of HA staining are presented in Figure 6E. Hyaluronic acid was seen throughout the ISTM, where the staining intensity was significantly greater than within sub-tendons (*p* < 0.001; Figure 6F).

### Collagen Type III Distribution

Typical images of collagen type III staining are presented in Figure 6I. There was a significant difference between the staining intensity of the ISTM and sub-tendon regions (*p* < 0.001; Figure 6J). Collagen type III appears to be predominately localized to the ISTM, with light staining also sparsely distributed throughout the sub-tendon regions between fibers.

### Lubricin Distribution

Typical images of lubricin staining are presented in Figure 6M. There was a significant difference between the staining intensity of the ISTM and STM regions (*p* < 0.001), where lubricin was predominately localized to the ISTM (Figure 6N). Lubricin was also evident within the sub-tendons, specifically at the interfaces between adjacent fibers.

### Elastin Distribution

Typical images of elastin staining are presented in Figure 6K. There was a significant difference between the staining intensity of the ISTM and sub-tendon regions (*p* < 0.001), with elastin predominately localized to the ISTM (Figure 6L). Staining for elastin was also present within sub-tendons, where it was localized to the interfaces between adjacent fibers.

### Assessor Variation

The overall Kappa score was 0.70, indicating a good agreement between the two blinded assessors. When focusing on the intra-observer agreement specifically, a Kappa score of 0.79 confirms consistency in scoring by each individual. There were no differences between the Kappa statistics for individual stains, or cellular parameters relating to the H&E staining.

## Discussion

The aim of this study was to investigate the capacity for force transmission between the GAS and SOL sub-tendons, testing the hypothesis that forces can be transmitted between the AT sub-tendons. We further aimed to assess the mechanical behavior and composition of the ISTM. We present the first data to clearly demonstrate force transmission between GAS to SOL sub-tendons. A direct analysis of ISTM mechanical behavior also demonstrated a non-linear loading curve, revealing an initial extended low stiffness region, followed by a swift rise in stiffness with increased applied force. Exploring specialization of the ISTM to enable this behavior, we provide the first investigation of protein distribution in the rat AT, which in support of our hypothesis, demonstrates a localization of elastin, lubricin, HA, and collagen type III to the ISTM.

This study is not without limitations. It is notable that sample preparation for mechanical testing was hindered by the small size of samples and the varying degrees of AT twist which led to a highly variable location of the ISTM. This made it difficult to carry out mechanical characterization in a repeatable manner, and meant that the overall number of samples were constrained. However, post-test analysis of the samples ensured that we could confidently pool data from isolated and non-isolated samples for analysis. Grip-to-grip strains are nearly always influenced by the gripping method, particularly in short samples such as a rodent AT. Here, the fixation of rings to the tendons may have led to increased compliance at the tendon-ring interface. However, surface markers were used in attempt to mitigate this error during strain analysis, so the applied strains could be correlated to those measured locally within sub-tendons. Whilst the sex of the animals for mechanical characterization was known, this was not the case for histological samples, and it is possible variation may exist in the composition of the AT among sexes. However, care was taken to remain semi-quantitative in the analysis of histological data, and recent work has also indicated that sex differences are largely insignificant in rodent ATs (Sarver et al., 2017). Indeed, the semi-quantitative nature of immunohistochemistry means care must also be taken in the interpretation. Staining intensity does not correlate to protein abundance, and is impacted by sample preparation. However, all samples were stained and imaged using a consistent protocol, and data focused on relative differences between different regions of the image, which would be less affected by such parameters. Whilst additional, quantitative approaches to analysis are necessary to determine relative amounts of proteins in the different regions of the AT, controls confirmed specificity of the stains, and good inter- and intra-observer agreement provide confidence in the reported outcomes.

Considering ISTM mechanics in light of the likely *in vivo* loading conditions, an extended low stiffness toe region of the ISTM loading curve would allow for sliding between the AT sub-tendons, and thus permit them a degree of mechanical independence. However, as the applied load increases, the rapid rise in ISTM stiffness would ensure force transmission between sub-tendons, and enable the AT to function as an integrated force transmitting structure. The capacity for inter-subtendon sliding in the AT has commonly led to the suggestion that the AT sub-tendons largely act independently under mechanical load. Such behavior could theoretically allow fibers within the GAS and SOL muscles to operate at independent lengths, which may be more optimal for their respective force production needs (Franz and Thelen, 2015; Franz et al., 2015). Indeed, two studies report negligible force transmission through the IFM in human (Haraldsson et al., 2008) and bovine AT (Purslow, 2009). Contrary to these reports, investigations into both equine and porcine flexor tendons have shown that whilst the IFM enables interfascicular sliding, it is also engaged in transmitting significant forces between adjacent fascicles (Thorpe et al., 2012, 2013b, 2015b; Kondratko-Mittnacht et al., 2015). The redistribution of load through the IFM similarly occurs in the rat tail tendon (Kondratko-Mittnacht et al., 2015). The current data provide further support to the importance of the ISTM in the rat AT in transmitting substantial forces between sub-tendons, enabling the AT to internally distribute stresses.

Our observations regarding the viscoelastic properties of the ISTM suggests greater hysteresis than seen in previous equine IFM tests, but comparable levels of percentage force reduction during the early stages of stress relaxation (Thorpe et al., 2015b), which may be related to the more complex, twisted structure of the AT or the complexity of the sample gripping arrangement. Despite this, it is evident the ISTM can effectively withstand and recover from cyclic loading. Recent studies indicate that aging reduces the elasticity of the IFM in the SDFT (Thorpe et al., 2015b), and rat tail tendon (Muench et al., 2019). Whether the same age-related changes occur in the ISTM of the AT is unknown, but such a response may be associated with the increased predisposition to AT injury with aging.

In support of our hypothesis, the ISTM demonstrated a distinctive protein composition and cellular organization, compared to that within sub-tendons, with collagen type III, elastin, HA, lubricin, and cellularity all enriched in the ISTM. In agreement with previous reports (Thorpe et al., 2015a, 2016b; Rowson et al., 2016), further analysis of the resident AT cells also revealed the nuclear morphology of the cells within the ISTM to be significantly rounder, which is likely to derive from the distinction between the physical cues imposed on the cells within each region (softer ISTM permits a more rounded shape, as opposed to the stiffer aligned collagen within sub-tendons restricting cell nuclei to aligning more toward the fiber direction).

Staining for decorin was not significantly different between the ISTM and sub-tendon regions of the rat AT. These results are in agreement with those observed in the equine SDFT, which have similarly shown staining for decorin throughout the IFM and fascicle regions (Kim et al., 2010; Thorpe et al., 2016a). Conversely, we found collagen type III to be significantly more enriched in the ISTM. Collagen type III is traditionally thought to be negligible in adult tendon unless tendinopathic (Maffulli et al., 2000; Eriksen et al., 2002; Södersten et al., 2013). However, whilst fascicles lose their collagen type III expression past development, the IFM has been shown to become significantly more enriched (Birk and Mayne, 1997), which is in line with our findings. These results may indicate a role for collagen III beyond that of repair, which we propose may be mechanical. Tissues with a high degree of elasticity, such as skin, blood vessels, and lung, demonstrate a significantly higher ratio of collagen type III:type I (Keene et al., 1987; Gelse et al., 2003), perhaps explained by the greater compliance of type III (Asgari et al., 2017). The endomysium around muscle fibers of GAS and SOL muscles in rat is similarly rich in collagen type III, where it is suggested to permit force transmission between muscle fibers (Kurose et al., 2006). It is possible, that this same mechanism also exists in the ISTM of the AT. Observed staining for type III collagen between the fibers of sub-tendons suggests it may also contribute the same mechanism at a fiber-level.

Elastin and lubricin were also highly localized to the ISTM, reflecting findings in the IFM of other energy storing tendons, where it is suggested lubricin enables interfascicular sliding, whilst elastin facilitates recoil (Ritty et al., 2002; Sun et al., 2006, 2009, 2015; Funakoshi et al., 2008; Kohrs et al., 2011; Grant et al., 2013; Fang and Lake, 2016; Thorpe et al., 2016a; Eekhoff et al., 2017; Godinho et al., 2017). Lubricin is a mucinous glycoprotein, which provides articular cartilage with boundary lubrication (Swann et al., 1977). Knockout studies have shown an absence of lubricin leads to an increase in interfascicular friction (Kohrs et al., 2011) and alterations in the viscoelastic properties of the fascicles (Reuvers et al., 2011). Elastin forms the core of elastic fibers, which are rich in tissues subjected to long-term repetitive loading [e.g., cardiovascular tissues (Li et al., 1998)], due to its ability to reversibly deform up to 100% strain with minimal energy loss (Gosline et al., 2002). Recent studies indicate elastin may also contribute to the mechanics of energy-storing tendons under both tension and shear, by facilitating the efficient recoil between fascicles (Grant et al., 2015; Fang and Lake, 2016; Eekhoff et al., 2017). Taken together, it is highly likely the localization of the lubricin and elastin in the ISTM is to support the elastic sliding and recovery of the ISTM as observed in this study.

It is notable that staining for lubricin was not homogeneous along the length of the ISTM, but far more prominent in the mid-portion and distal regions of the AT, which is directly in line with observations made in the human AT (Sun et al., 2015). Spiralization of the Achilles sub-tendons generates an area of concentrated stress ∼3–6 cm above the calcaneal insertion in humans (Doral et al., 2010), which correlates to the region of the tendon with the narrowest CSA (Kongsgaard et al., 2005), and highest concentration of lubricin (Sun et al., 2015). While we did not analyze lubricin in quantitative amounts, we established that the region of intense staining in the rat Achilles ISTM correlated to the same approximate region as reported in the human AT. Interestingly, we found that the concentration of HA mirrored that of lubricin, with particular enrichment in the distal ISTM. Hyaluronic acid has been identified in the compressive regions of flexor tendons (Robbins and Vogel, 1994; Rees et al., 2005; Vogel and Peters, 2005), but also within the sheath, where it primarily serves a tribological role (Hagberg et al., 1992; Uchiyama et al., 1997). Co-localization of HA and lubricin in the ISTM may indicate both play similar mechanical roles in tendon, potentially protecting against increased compressive and shear forces in this region. However, a recent study revealed that a synergistic relationship between HA and lubricin was fundamental in providing articular cartilage with its remarkably low friction lubrication (Bonnevie et al., 2015), providing support to the hypothesis that these components may also work together in tendon to facilitate sub-tendon sliding. It is notable that the region of concentrated lubricin and HA also correlates to the region of the AT most prone to tendinopathy, which may indicate an association between sub-tendon sliding and injury (Theobald et al., 2005; Shim et al., 2014).

## Conclusion

This study is the first to investigate ISTM mechanical behavior and demonstrate force transmission between AT sub-tendons. Non-linear mechanical behavior of the ISTM combines an initial low stiffness region with a region of high linear stiffness. Together, these data reveal that the ISTM is capable of enabling non-uniform AT loading whilst still ensuring force transmission occurs between the AT sub-tendons. An analysis of ISTM composition highlights localization of HA, lubricin, and elastin to the ISTM region, proteins likely to play a role in facilitating the sliding and recovery of the ISTM. Our results provide important advances into the understanding of AT structure and function. Further studies into the effects of aging on force transmission may reveal the factors which predispose the AT to injury, thus assisting in the development of appropriate preventative therapies.

## Data Availability Statement

All datasets generated for this study are included in the article/supplementary material.

## Ethics Statement

Ethical review and approval was not required for the animal study because tissue taken as waste tissue from other unrelated studies.

## Author Contributions

HM conceived and designed the experiments and edited the manuscript. HS assisted with the experimental design and edited the manuscript. CG performed the immunohistochemistry, analyzed all the data, and drafted the manuscript. JC, GB, and WN performed the mechanical experiments. All authors contributed to the article and approved the submitted version.

## Conflict of Interest

The authors declare that the research was conducted in the absence of any commercial or financial relationships that could be construed as a potential conflict of interest.

## Footnotes

1 http://vassarstats.net/kappa.html
